# The FGFR1 N546K mutation confers resistance to pemigatinib in MLN-*ZMYM2::FGFR1*

**DOI:** 10.1038/s41375-026-02890-w

**Published:** 2026-02-20

**Authors:** Khalid Shoumariyeh, Stefan Haug, Juliana Schwaab, Julie K. Pfeil, Jannis Stappenbeck, Annette Schmitt-Graeff, Nicole Naumann, Greta Waltemode, Robert Zeiser, Cornelius Miething, Wolf-Karsten Hofmann, Manja Meggendorfer, Sven Diederichs, Justus Duyster, Andreas Reiter

**Affiliations:** 1 Department of Medicine I, Medical Center – University of Freiburg, Faculty of Medicine, University of Freiburg, Freiburg, Germany; 2 German Cancer Consortium (DKTK), Partner Site Freiburg, a partnership between DKFZ and University Medical Center Freiburg, Freiburg, Germany; 3 Institute of Epidemiology and Prevention, Faculty of Medicine and Medical Center - University of Freiburg, Freiburg, Germany; 4 University Hospital Mannheim, Heidelberg University, Mannheim, Germany; 5 Division of Cancer Research, Department of Thoracic Surgery, Medical Center - University of Freiburg, Faculty of Medicine, University of Freiburg, Freiburg, Germany; 6 University of Freiburg, Freiburg, Germany; 7 MLL Munich Leukemia Laboratory, Munich, Germany

**Keywords:** Leukaemia, Leukaemia

## To the Editor

Myeloid/lymphoid neoplasms with fibroblast growth factor receptor 1 (*FGFR1*) gene fusions (MLN-*FGFR1*) are rare haematological neoplasms characterized by an aggressive clinical course and poor prognosis [1]. In the World Health Organization (WHO) and the International Consensus Classification (ICC) of myeloid and lymphoid neoplasms, MLN-*FGFR1* represents a specific subtype within the category “myeloid/lymphoid neoplasms with eosinophilia and tyrosine kinase fusion genes” (MLN-TK [WHO] or M/LN-eo-TK [ICC]) [2, 3].

The genetic hallmark of MLN*-FGFR1* are reciprocal translocations involving chromosome band 8p11 that result in the fusion of variable partner genes with *FGFR1*, most commonly *ZMYM2* (MLN-*ZMYM2::FGFR1*), t(8;13)(p11.2;q12), and *BCR* (MLN-*BCR::FGFR1)*, t(8;22)(p11.2;q11.2), leading to ligand-independent constitutive activation of the FGFR1 tyrosine kinase domain (TKD) through N-terminal dimerization domains of the partner genes. To date, 20 *FGFR1* fusion partners have been identified [4]. The initial clinical phenotype is often characterized by a myeloid neoplasm in chronic phase (CP) variably associated with eosinophilia and occasionally accompanied by an extramedullary disease (EMD, histologically manifesting as T-cell lymphoma, rarely B-cell lymphoma or myeloid sarcoma) or a primary blast phase (BP) in the bone marrow (BM) of myeloid, lymphoid, or mixed phenotype. CP disease generally shows rapid progression into secondary BP within 1–2 years [4].

There are only a few reports describing usually partial and non-durable responses to currently available TKIs [4, 5]. Moreover, durable complete morphologic or molecular remissions are only rarely achieved following intensive chemotherapy for lymphomas or acute leukemias in patients with BP. In eligible patients, early allogeneic hematopoietic cell transplantation (allo-HCT) during CP or following remission induction after intensive chemotherapy is therefore recommended for all patients to improve long-term outcome [4, 6].

Pemigatinib (INCB054828) is a selective, orally available, reversible FGFR1-3 inhibitor, initially approved for advanced, treatment-refractory *FGFR2*-rearranged cholangiocarcinoma. In phase 2, the open-label FIGHT-203 study, which enrolled 47 patients with MLN-*FGFR1*, pemigatinib induced an overall complete response in 74% of patients (96% in CP and 44% in BP) and a 73% complete cytogenetic response (CCyR) rate. The median duration of complete response was not reached (27.9 months to not reached) [7]. Based on the interim results of the FIGHT-203 study, the FDA granted approval for pemigatinib in 2022 for relapsed or refractory MLN-*FGFR1*. However, no mechanisms of acquired resistance to pemigatinib in MLN-*FGFR1* have been described to date.

Here, we report an MLN-*ZMYM2::FGFR1* in CP and concurrent myeloid EMD at multiple sites. The patient achieved a rapid but short-term response to pemigatinib, with subsequent progression of a myeloid EMD associated with the acquisition of a TKD mutation in *FGFR1* exon 12 affecting the hotspot amino acid residue 546 (FGFR1 N546K).

**Fig. 1:**
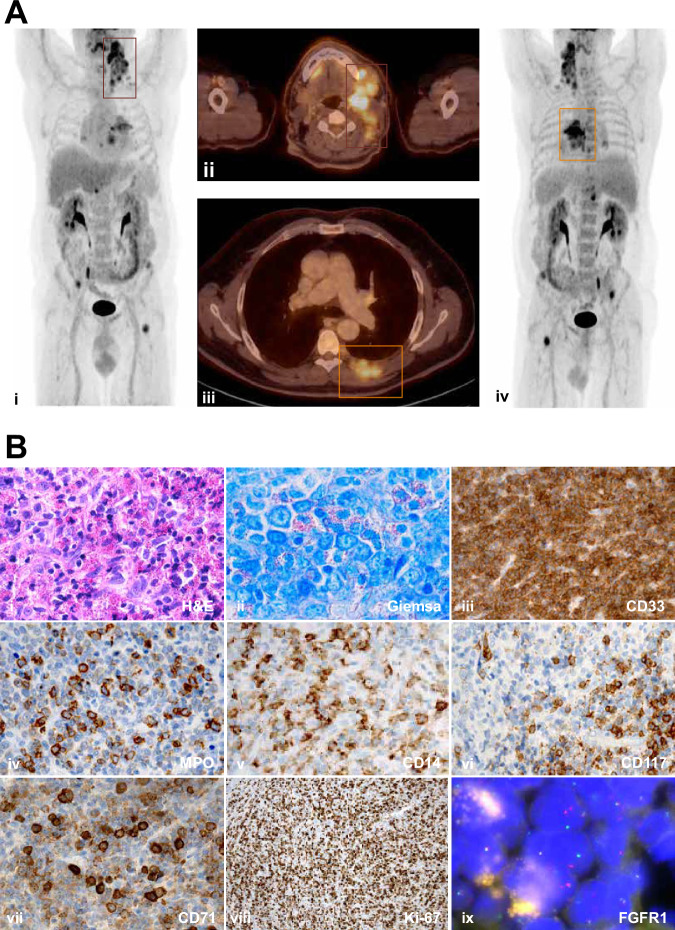
Radiologic and histologic features at diagnosis. **A** PET-CT imaging. i, ii Representative PET-CT images demonstrating FDG-avid hypermetabolic cervical lymphadenopathy (red squares). iii, iv PET-CT images highlighting a hypermetabolic lesion of the left dorsal thoracic wall consistent with myeloid sarcoma (orange squares). **B** Morphologic, immunohistochemical, and cytogenetic characterization. i, ii Histopathology showing sheets of immature myeloid cells with prominent nucleoli and numerous eosinophil precursors. iii Strong CD33 expression by immunohistochemistry. iv Subset of blasts positive for myeloperoxidase (MPO). v CD14 expression indicating partial myelomonocytic/monoblastic/histiocytic differentiation. vi CD117 expression marking blasts and mast cells. vii CD71 positivity, suggesting an erythroid lineage component. viii Ki-67 immunoreactivity indicating high proliferative activity. ix FISH using a break-apart probe demonstrating *FGFR1* rearrangement. CD cluster of differentiation, FDG fluorodeoxyglucose, FGFR1 fibroblast growth factor receptor 1, FISH fluorescent in situ hybridization, PET-CT positron emission tomography-computed tomography.

The 64-year-old male patient presented with a two-month history of jaw pain and progressive cervical lymphadenopathy. He had no significant past medical history. A positron emission tomography (PET) scan demonstrated hypermetabolic hilar, mediastinal, and cervical lymphadenopathy (Fig. 1Ai, ii), along with a metabolically active soft tissue lesion on the left dorsal thoracic wall (Fig. 1Aiii, iv). Bone involvement was noted in the pelvis and the left dorsal sixth rib. Blood counts and differential were within normal ranges. A cervical lymph node biopsy revealed infiltration by myeloid blasts (CD33+, CD34+, CD117+, MPO+, CD71+) accompanied by marked eosinophilia (Fig. 1Bi–viii). Cytogenetics from BM aspirate cells revealed a t(8;13)(p11;q12)[16],46,XY[4] karyotype. Fluorescent in situ hybridization (FISH) confirmed a rearrangement of *FGFR1* (Fig. 1Bix), and reverse-transcriptase polymerase chain reaction (RT-PCR) identified an in-frame *ZMYM2::FGFR1* fusion transcript (*ZMYM2* exon 16 (ENST00000610343.5) fused to *FGFR1* exon 10 (ENST00000447712.7); ZMYM2-Ex16-F1: GACAGAATATGTTCCAGTGCCT, FGFR1_Ex10-R1: AGCTCATA CTCAGAGAMCCC, ZMYM2-Ex16-F2: CTGTGCCTGTGTATATCCCRGTT, FGFR1_Ex10-R2: GTGATGGCCGAACCAGAAGMA). The final diagnosis was MLN-*ZMYM2::FGFR1* with myeloid EMD at multiple sites.

After informed consent, the patient was enrolled in the phase 2 FIGHT-203 study [7]. Pemigatinib 13.5 mg once daily (two weeks on/one week off) was well tolerated, and induced a rapid, complete haematological response and CCyR. However, *ZMYM2::FGFR1* fusion transcripts remained detectable by droplet digital polymerase chain reaction (ddPCR).

Following the third cycle, he developed left-sided thoracic pain and dyspnea. Imaging studies revealed left-sided pleural effusion and osteolytic destruction of the fifth and sixth rib consistent with progressive myeloid sarcoma. Biopsy of the soft tissue mass confirmed CD34+ blasts and a rearrangement of *FGFR1*.

**Fig. 2:**
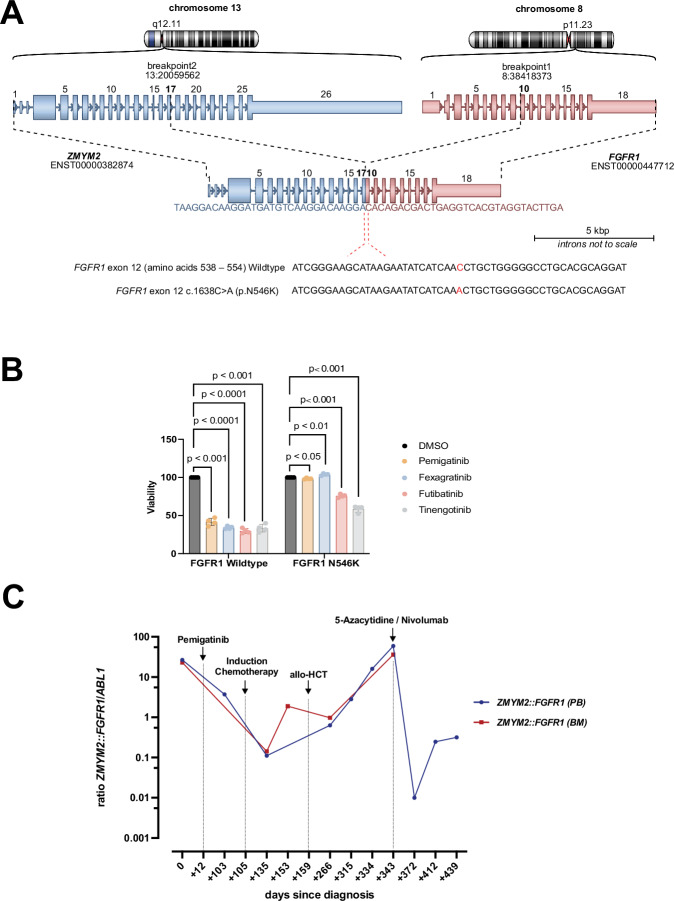
Molecular profiling of *FGFR1* at clinical resistance, sensitivity of FGFR1 N546K to FGFR1 inhibitors, and longitudinal monitoring of the *ZMYM2::FGFR1* fusion. **A** *ZMYM2::FGFR1* fusion and *FGFR1* mutation. Schematic representation of the breakpoints in *ZMYM2* and *FGFR1* involved in the fusion transcript. The *FGFR1* exon 12 sequence (amino acids 538-554) is shown, highlighting the c.1638C>A point mutation (red) identified in DNA isolated from the progressive thoracic wall myeloid sarcoma. **B** Sensitivity of the FGFR1 N546K mutation to FGFR1 inhibitors. Cell viability of NCI-H1581 cells stably transduced with either FGFR1 wild-type or FGFR1 N546K mutant following treatment with multiple FGFR inhibitors. The mean of four independent biological replicates is depicted ± standard error of the mean. Statistical significance calculated by a two-sided Student’s t-test is indicated. **C** Longitudinal monitoring of *ZMYM2::FGFR1* transcript levels. Droplet digital PCR (ddPCR) tracking of *ZMYM2::FGFR1* transcript levels in bone marrow (BM) and peripheral blood (PB) over the disease course. BM bone marrow, cDNA complementary DNA, ddPCR droplet digital polymerase chain reaction, FGFR1 fibroblast growth factor receptor 1, PB peripheral blood, ZMYM2 Zinc Finger MYM-Type Containing 2.

To explore potential resistance mutations to pemigatinib, Sanger sequencing (FGFR_P1_F: CTGACTCCAGTGCATCCATGAAC; FGFR_P1_R: TGCACTTC TTGGAGGCCAGATAC; FGFR_P2_F: GAATACTGCTACAACCCCAGCC; FGFR_P2_R: CTGAGGAGCACGTAGAGCTC) of the FGFR1 TKD using cDNA from the progressive myeloid sarcoma was performed. A missense mutation in *FGFR1* exon 12 was identified (*FGFR1* Chr8: g.38417331G>T (GRCh38.p13), c.1638C>A, ENST00000447712.7 (NM_023110.3), p.Asn546Lys), resulting in an asparagine-to-lysine substitution at position 546 (p.N546K) (Fig. 2A).

FGFR1 N546K has been reported as an activating mutation in central nervous system tumors and Ewing’s sarcoma [8–10]. It enhances FGFR1 KD autophosphorylation and promotes cellular transformation [11]. In contrast to the FGFR1 V561M gatekeeper mutation, which sterically hinders ATP-competitive inhibitor binding, the N546K mutation confers resistance to multiple TKIs (e.g., ponatinib, dovitinib, PD173074, and BGJ-398) by increasing FGFR1’s affinity to ATP [12]. Structural analyses of the homologous FGFR2 N549D/K mutations suggest disruption of stabilizing hydrogen bonds, which shift the kinase into an active conformation [13].

In cell viability assays (Supplementary Materials), the FGFR1 N546K mutation markedly reduced sensitivity to the FGFR1 inhibitors pemigatinib and fexagratinib (pemigatinib: WT vs. N546K, −58% vs. −2%; fexagratinib: WT vs. N546K, −66% vs. 4%), with N546K-expressing cells maintaining substantially higher viability than FGFR1 wild-type controls (Fig. 2B). In contrast, futibatinib and tinengotinib retained appreciable activity against the N546K variant (futibatinib: WT vs. N546K, −70% vs. −24%; tinengotinib: WT vs. N546K, −67% vs. −41%), although their efficacy remained reduced relative to wild-type cells (Fig. 2B). Collectively, these findings demonstrate that FGFR1 N546K confers resistance to pemigatinib and fexagratinib and identify futibatinib and tinengotinib as potential therapeutic options capable of partially overcoming the FGFR1 N546K resistance mutation. Notably, these experiments were performed using wild-type and mutant FGFR1 rather than the ZMYM2::FGFR1 fusion protein; therefore, the observed inhibitor sensitivities may not fully reflect the fusion-specific signaling context or drug responsiveness in MLN-*ZMYM2::FGFR1*, warranting further investigation in fusion-based model systems.

The patient was taken off study and received local radiotherapy (5 × 4 Gy) to the progressive myeloid sarcoma, followed by induction chemotherapy (mitoxantrone, idarubicin, etoposide, cytarabine). Subsequently, he underwent HLA-matched allo-HCT with a reduced-intensity conditioning regimen (fludarabin, treosulfan, thiotepa) and received cyclosporine and mycophenolate mofetil as graft-versus-host disease (GvHD) prophylaxis. Following allo-HCT, the patient achieved a complete response and remained in a CCyR (Fig. 2C).

Six months after allo-HCT, he developed left leg swelling. Imaging studies and ddPCR (*ZMYM2::FGFR1* Fwd: CTATCCCTGTGCCTGTGTATATC; *ZMYM2::FGFR1*_Ex10 probe: 5’-FAM-TGCTGACTC/ZEN/CAGTGCATCCATGAA-3IABkFQ; *ZMYM2::FGFR1* Rev: GATGGCCGAACCAGAAGAA, Fig. 2C) confirmed clinical and molecular relapse. To leverage the graft-versus-leukemia effect, he received 5-azacytidine followed by nivolumab, which led to a marked decline of *ZMYM2::FGFR1* transcript levels in the peripheral blood after the first cycle and a clinical response (Fig. 2C) [14]. However, he developed severe refractory gastrointestinal GvHD and succumbed to his disease 19 months after the initial diagnosis and 12 months post-allo-HCT.

In summary, we identified an acquired FGFR1 N546K TKD mutation conferring resistance to pemigatinib in a patient with MLN-*ZMYM2::FGFR1*. While the pivotal FIGHT-203 trial established pemigatinib as an effective treatment option with high response rates and durable remissions, particularly in MLN-*FGFR1* in CP, our findings illustrate that resistance mutations such as FGFR1 N546K may emerge [7]. Our observation highlights the importance of screening for TKD mutations in patients with MLN-*FGFR1* on TKI at the time of disease progression, similar to *BCR::ABL1-*positive CML. Further research is required to define the frequency of FGFR1 TKD mutations and to assess the efficacy of alternative FGFR1 inhibitors, e.g., futibatinib or tinengotinib, to overcome TKD-associated resistance [15].

## Supplementary information

Supplementary Materials and Methods
