# Supplementary material for: The FGFR1 N546K mutation confers resistance to pemigatinib in MLN-ZMYM2::FGFR1
Source: Leukemia. 2026 Feb 20;40(4):837–40. doi: 10.1038/s41375-026-02890-w (PMC13056580; doi:10.1038/s41375-026-02890-w)
Supplement: Supplementary file 1 — Supplementary Materials and Methods [file 41375_2026_2890_MOESM1_ESM.pdf]

## Supplementary Materials and Methods

### Cell Lines and Constructs

The FGFR inhibitor–sensitive lung cancer cell line NCI-H1581 (ATCC - CRL-5878) was used for all experiments. NCI-H1581 cells were maintained in DMEM:F12 1:1 + GlutaMax + 10% FBS. All media supplements were purchased from Sigma / Merck KGaA. Cells were stably transduced with either wildtype FGFR1 (FGFR1 WT) or the FGFR1 N546K mutation using pHAGE lentiviral constructs encoding the respective cDNAs and selected by puromycin as previously described.[1] NCI-H1581 cells were cultured under standard conditions (37°C, 5% $CO_2$) and routinely tested to be free of mycoplasma contamination.

### Drug Compounds

The four FGFR1 inhibitors pemigatinib (MedChemExpress, HY-109099-5mg), fexagratinib (MedChemExpress, HY-13330-10mg), futibatinib (MedChemExpress, HY-109099-5mg), and tinengotinib (MedChemExpress, HY-145601-10mg) were evaluated for their activity against FGFR1 WT and FGFR1 N546K mutant cells. All compounds were commercially obtained, prepared according to manufacturer recommendations, and diluted in DMSO, which served as the vehicle control. Drug concentrations were selected based on previously determined $IC_{50}$/$ED_{50}$ values in NCI-H1581 cells: pemigatinib (20 nM), fexagratinib (50 nM), futibatinib (20 nM), and tinengotinib (200 nM).

### Cell Viability Assay

Cell viability was assessed using the CellTiterGlo® luminescent cell viability assay (Cat.# G7570, Promega, Germany) in a 96-well plate format. FGFR1 WT and FGFR1 N546K cells were plated and allowed to adhere overnight before drug treatment. Cells were exposed to the indicated FGFR inhibitors for 72 hours, with media and drug replenishment after 48 hours. Luminescence was measured according to the manufacturer's protocol but with the reagent diluted 1:4 with PBS prior to use, and viability was normalized to DMSO-treated controls (set

to 100% for each biological replicate). Each experimental condition included four biological replicates, with each biological replicate representing the mean of four technical replicates.

## Statistical Analysis

Statistical analyses were conducted using Student's t-test following an F-test to assess variance equality. Significance thresholds were defined as $p < 0.05$, $p < 0.01$, $p < 0.001$, and $p < 0.0001$. Comparisons were made between DMSO and each drug treatment condition independently for FGFR1 WT and FGFR1 N546K cells.

## SUPPLEMENTARY REFERENCES

1 Tangermann C, Ghosh A, M Ziegler, Faccinetti F, Stappenbeck J, Carus Sahin Y *et al.* Saturation mutagenesis identifies activating and resistance-inducing FGFR kinase domain mutations. *Nature Genetics* 2025. doi:10.1038/s41588-025-02431-8.
